# Relationship between burnout and career choice regret among Chinese neurology postgraduates

**DOI:** 10.1186/s12909-019-1601-3

**Published:** 2019-05-22

**Authors:** Lu Tian, Juncai Pu, Yiyun Liu, Xiaogang Zhong, Siwen Gui, Xuemian Song, Shaohua Xu, Xinyu Zhou, Haiyang Wang, Wei Zhou, Jianjun Chen, Peng Xie

**Affiliations:** 1 School of Public Health and Management, Chongqing Medical University, Chongqing, 400016 China; 2 Institute of Neuroscience, Chongqing Medical University, Chongqing, 400016 China; 3 Department of Neurology, The First Affiliated Hospital of Chongqing Medical University, Chongqing, 400016 China; 4 College of Biomedical Engineering, Chongqing Medical University, Chongqing, 400016 China; 5 China Neurologist Association of Chinese Medical Doctor Association, Beijing, 100010 China

**Keywords:** Neurology, Postgraduates, Burnout, Career choice regret, China

## Abstract

**Background:**

In China, the shortage of doctors leads to stressful clinical work and increasing turnover. Medical students undergoing postgraduate specialty training will be the country’s medical workforce in the coming decades, but are also subject to high workloads and academic pressure. This may have significant implications for burnout and career choice regret. Despite the importance of burnout and career choice regret, the status and relationship of these aspects in Chinese neurology postgraduates are largely unexplored, and associated factors remain unknown.

**Methods:**

This study investigated the prevalence of and factors influencing burnout and career choice regret among neurology postgraduates in China. We conducted a national cross-sectional study of Chinese neurology postgraduates. Data were collected using a self-administered questionnaire that covered demographic information, the Maslach Burnout Inventory, and additional item to assess career choice regret.

**Results:**

Of 4902 neurology postgraduates, 2008 returned completed questionnaires (response rate 41%). After excluding incomplete questionnaires, data for 1814 participants were analyzed. In total, 83.6% of participants had experienced symptoms of burnout, and 46.6% reported career choice regret. Binary logistic regression analysis showed postgraduate entrance examination scores, marital status, and having children were associated with burnout (all *P* <  0.05). Career choice regret was the strongest risk factor for burnout (odds ratio [OR] = 3.17, 95% confidence interval [CI] 2.33–4.32). Multiple logistic regression showed postgraduates with shorter work or study hours per week (OR = 0.64, 95% CI 0.47–0.88) had a low risk for career choice regret, whereas married participants (OR = 1.54, 95% CI 1.07–2.20) had a high risk for career choice regret. No symptoms of burnout (OR = 0.33, 95% CI 0.24–0.45) was also associated with a low risk for career choice regret.

**Conclusions:**

Burnout symptoms and career choice regret are prevalent among neurology postgraduates in China. Career choice regret is an important predictor of burnout. Further research on reducing burnout and career choice regret among neurology postgraduates is needed.

**Electronic supplementary material:**

The online version of this article (10.1186/s12909-019-1601-3) contains supplementary material, which is available to authorized users.

## Background

Burnout is a prolonged physical and mental response to chronic emotional and interpersonal job-related stressors [1]. It is characterized by emotional exhaustion, depersonalization, and low personal accomplishment, and has been an important research topic in psychology, organizational behavior, and human resource management for nearly 30 years [2, 3]. Individuals engaged in work that requires more personal sacrifice, particularly medical workers, are at high risk for burnout [4]. The high prevalence of burnout among physicians has become a global issue. For example, burnout was reported by 54.4% of physicians in the US [5], 50% of physicians in Brazil [6], 72% of psychiatrists in Japan [7], and 60.6% of physicians in China [8]. Moreover, burnout may lead to poor patient care and reduced job satisfaction [9].

Medical postgraduates or interns often work long hours and have heavy workloads, which may increase the risk for burnout and career choice regret. The incidence of burnout was 20% among medical interns in Mexico [10], 52.8–76% in US medical students [11, 12], and 45.2% in US resident physicians [13]. In China, medical postgraduates are resident physicians or junior researchers who receive standardized training for 3 years [14, 15]. The incidence of burnout in this population is reported to range from 38.4–51.3% [16, 17]. Burnout is also associated with career choice regret, unprofessional conduct, and even suicidal ideation [13, 18]. Burnout was found to be the single greatest predictor of career choice regret among US resident physicians and surgeons [19]. Other studies also showed that burnout and career choice regret were common among doctors and medical postgraduates. For example, previous studies reported 58.1% of neurologists regretted becoming a doctor [20] and 62% of medical students regretted choosing to study medicine [21]. However, factors associated with burnout and career choice regret vary among different populations. Individual characteristics, organizational factors, and sub-specialty choice may be determinants of burnout and career choice regret among medical students [13, 22].

Previous surveys suggest that neurology is a specialty with high levels of job-related burnout and low levels of satisfaction [23, 24]. The workforce demand for neurologists has increased sharply in recent years with population aging. A survey in the US estimated the shortfall of neurologists was approximately 11% in 2012, but would increase to 19% by 2025 [25]. Career satisfaction among neurologists has also declined in recent years [26], which suggests employment motivation among medical postgraduates may be important in addressing the shortage of doctors. However, more research is needed to identify determinants of burnout and career choice regret for Chinese neurology postgraduates. Alleviating the symptoms of burnout and reducing career choice regret to maintain neurology postgraduates’ original intention of studying medicine may decrease the turnover rate and ensure the future supply and quality of neurologists.

Despite the widely acknowledged importance of burnout and career choice regret [13, 27], the status and relationship of these two aspects among Chinese neurology postgraduates are largely unexplored. In this national study, we examined the status of and factors associated with burnout and career choice regret among Chinese neurology postgraduates. Based on our findings, we proposed some suggestions that may be helpful to inform employment measures.

## Methods

### Study designs, setting, and participants

This survey was conducted by the China Neurologist Association from September 2014 to March 2015. The study design was adapted from our previous study [20]. In brief, with the assistance of local neurologist associations, neurology postgraduates were invited to complete a self-administered questionnaire. Data collection and analysis were performed by independent researchers blinded to the experiment design.

### Standard protocol approval and participant consent

The cover letter for the questionnaire introduced the purpose of the survey, which was to explore the status of and factors potentially associated with burnout and career choice regret. The cover letter also stated that participation was voluntary and anonymous, and personal privacy would not be violated in the study processes. Consent was assumed for any participant who returned a completed survey. Ethics approval was granted by the Ethics Committee of Chongqing Medical University.

### Survey questionnaire

The survey questionnaire contained four parts. Part 1 covered demographic variables, including gender, academic year, age, degree type, postgraduate entrance examination score, hours worked or studied per week, hours slept per day, marital status, whether the respondent had children, and work type (part-or full-time). Part 2 comprised the Maslach Burnout Inventory [28]. This instrument measures three domains of burnout (emotional exhaustion, depersonalization, and personal accomplishment) using a seven-point Likert-type scale (range 0–6). We considered participants to have high levels of burnout if they scored more than 27 points for emotional exhaustion or more than 10 points for depersonalization [20, 29]. Part 3 included a question investigating career choice regret: “If you could go back, would you choose to become a doctor again?” Response options were ‘no’, ‘neutral’ and ‘yes’; responses of ‘no’ indicated career choice regret. This question was used to assess career choice regret in previous studies [13, 19]. Part 4 included three additional questions: “Will you choose to become a doctor after graduation?”, “What do you think of the current medical environment?” and “Have you ever considered dropping out?”

### Statistical analysis

All statistical analyses were performed with SPSS version 21.0 (IBM Corp, Armonk, NY, USA). Associations between variables were evaluated using univariate analysis with a chi-square test for categorical variables. Binary logistic regression analysis was used to verify the factors affecting burnout. We fitted three logistic regression models. Model 1 was adjusted for all demographic variables, Model 2 for career choice regret, and Model 3 for demographic variables and career choice regret. Multicollinearity analysis was used to test collinearity among variables when the tolerance was ≤0.1 or the variance inflation factor was ≥10. Three methods (enter model, backward elimination method, and forward elimination method) were used to select significant independent variables in the logistic models. Multiple logistic regression analysis was used to identify factors potentially associated with career choice regret. For the dependent variable (career choice regret), the category ‘without career choice regret’ was set as the reference category. Demographic variables and burnout were independent variables. A *P*-value below 0.05 was considered statistically significant.

## Results

In March 2015, 4902 questionnaires were distributed, and 2008 collected from 249 hospitals (response rate 41.0%). We excluded 194 invalid questionnaires because of missing data for the Maslach Burnout Inventory, leaving 1814 questionnaires for analysis.

### Demographic characteristics

Table 1 shows the characteristics of the participating neurology postgraduates. Overall, 67.1% of participants were female, 68.5% were in clinical practice with the remainder in academic positions, and 85.7% had a master’s degree. Around half of the participants reported a family income less than 5000 RMB per month, and 40.2% worked more than 55 h per week. Most participants were unmarried (83.1%) and 67.5% did not have children.

**Table 1:** Demographic characteristics associated with burnout

Characteristics	N	%	Burnout		*P*
			Without	With	
Gender					
Male	593	32.7	112 (37.6)	481 (31.8)	0.05
Female	1218	67.1	186 (62.4)	1032 (68.2)	
Academic year					
First-year, master’s degree	391	21.6	74 (25.8)	317 (21.3)	0.10
Second-year, master’s degree	554	30.5	77 (26.8)	477 (32.1)	
Third-year, master’s degree	609	33.6	109 (38.0)	500 (33.6)	
First-year, doctor’s degree	79	4.4	10 (3.5)	69 (4.6)	
Second-year, doctor’s degree	73	4.0	11 (3.8)	62 (4.2)	
Third-year, doctor’s degree	68	3.7	6 (2.1)	62 (4.2)	
Degree type					
Clinical practice	1243	68.5	209 (71.3)	1034 (69.8)	0.59
Academic practice	532	29.3	84 (28.7)	448 (30.2)	
Family income (yuan per month)					
< 5000	901	49.7	154 (51.9)	747 (49.6)	0.88
5000–10,000	603	33.2	95 (32.0)	508 (33.7)	
10,000–15,000	176	9.7	27 (9.1)	149 (9.9)	
> 15,000	124	6.8	21 (7.1)	103 (6.8)	
Scores of postgraduate entrance examination					
< 300	221	12.2	55 (21.5)	166 (13.0)	< 0.01
300–330	698	38.5	93 (36.3)	605 (47.4)	
330–360	422	23.3	75 (29.3)	347 (27.2)	
> 360	192	10.6	33 (12.9)	159 (12.5)	
Hours worked or studied per week (h)					
< 35	132	7.3	28 (9.5)	104 (6.9)	0.02
35–45	428	23.6	75 (25.4)	353 (23.4)	
45–55	512	28.2	96 (32.5)	416 (27.6)	
> 55	732	40.4	96 (32.5)	636 (42.1)	
Hours slept per day (h)					
< 6	224	12.3	33 (11.1)	191 (12.6)	0.25
6–8	1462	80.6	238 (79.9)	1224 (80.8)	
8–10	126	6.9	27 (9.1)	99 (6.5)	
Marital status					
Married	302	16.6	65 (21.8)	237 (15.7)	0.01
Unmarried	1507	83.1	233 (78.2)	1274 (84.3)	
Whether have children					
Without	1224	67.5	222 (74.5)	1002 (66.2)	0.01
Have	588	32.4	76 (25.5)	512 (33.8)	
Whether to work part-time once					
Without	1116	61.5	187 (63.6)	929 (61.6)	0.51
Have	687	37.9	107 (36.4)	580 (38.4)	

### Prevalence and univariate analysis of burnout and career choice regret

**Fig. 1:**
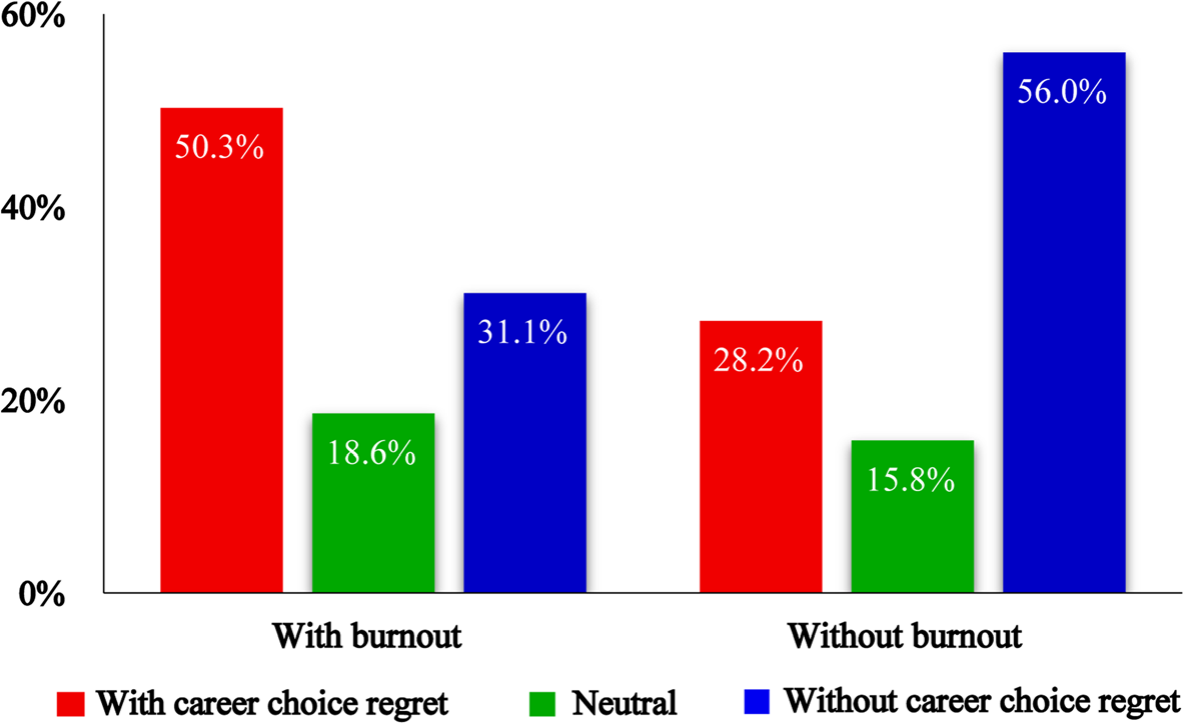
The distribution of career choice regret among postgraduates with burnout

Table 2 summarizes the prevalence of burnout and career choice regret. In general, 83.6% of participants had symptoms of burnout (high emotional exhaustion or depersonalization scores). In terms of career choice regret, 46.6% said that they would not choose to be doctor again, and 18.1% were unsure. Half of the participants with symptoms of burnout had career choice regret (Fig. 1). In addition, 7.3% of participants said they did not want to be a doctor when they graduated and 17.6% had thought about dropping out at least once. Only 2.9% thought that the current medical environment was good. In Table 1, burnout was associated with postgraduate entrance examination score, hours worked or studied per week, marital status, and having children (all *P* <  0.05). In the univariate analysis (Table 3), career choice regret was associated with academic year, degree type, family income, hours slept per day, and marital status (all *P* <  0.05).

**Table 2:** Prevalence of burnout, career choice regret and responses to 3 other questions

Characteristics	N (%)
Burnout	
With	1516 (83.6%)
Without	298 (16.4%)
Career choice regret	
With	845 (46.6%)
Neutral	329 (18.1%)
Without	637 (35.1%)
Wished to be a doctor when graduate	
No	132 (7.3%)
Neutral	360 (19.8%)
Yes	1314 (72.4%)
View on current medical environment	
Good	52 (2.9%)
Neutral	711 (39.2%)
Poor	1043 (57.5%)
Considered dropping out once	
Yes	319 (17.6%)
No	1477 (81.4%)

**Table 3:** Demographic characteristics associated with career choice regret

Characteristics	Career choice regret			
	With	Neutral	Without	*P*
Gender				
Male	266 (44.9%)	112 (18.9%)	215 (36.3%)	0.57
Female	577 (47.5%)	217 (17.9%)	421 (34.7%)	
Academic year				
First-year, master’s degree	167 (42.7%)	63 (16.1%)	161 (41.2%)	< 0.01
Second-year, master’s degree	251 (45.5%)	120 (21.7%)	181 (32.8%)	
Third-year, master’s degree	295 (48.5%)	103 (16.9%)	210 (34.5%)	
First-year, doctor’s degree	29 (36.7%)	21 (26.6%)	29 (36.7%)	
Second-year, doctor’s degree	41 (56.2%)	12 (16.4%)	20 (27.4%)	
Third-year, doctor’s degree	40 (58.8%)	6 (8.8%)	22 (32.4%)	
Degree type				
Clinical practice	600 (48.4%)	210 (16.9%)	430 (34.7%)	0.03
Academic practice	225 (42.3%)	114 (21.4%)	193 (36.3%)	
Family income (yuan per month)				
< 5000	435 (48.4%)	133 (14.8%)	330 (36.7%)	< 0.01
5000–10,000	275 (45.6%)	101 (16.7%)	227 (37.6%)	
10,000–15,000	76 (43.2%)	51 (29.0%)	49 (27.8%)	
> 15,000	51 (41.1%)	43 (34.7%)	30 (24.2%)	
Scores of postgraduate entrance examination				
< 300	99 (45.2%)	34 (15.5%)	86 (39.3%)	0.51
300–330	335 (48.1%)	126 (18.1%)	236 (33.9%)	
330–360	185 (43.8%)	88 (20.9%)	149 (35.3%)	
> 360	86 (44.8%)	34 (17.7%)	72 (37.5%)	
Hours worked or studied per week (h)				
< 35	62 (47.0%)	18 (13.6%)	52 (39.4%)	0.14
35–45	193 (45.2%)	70 (16.4%)	164 (38.4%)	
45–55	225 (26.8%)	102 (31.3%)	185 (29.2%)	
> 55	361 (49.5%)	136 (18.6%)	233 (31.9%)	
Hours slept per day (h)				
< 6	122 (54.7%)	44 (19.7%)	57 (25.6%)	< 0.01
6–8	671 (46.0%)	253 (17.3%)	536 (36.7%)	
8–10	50 (39.7%)	32 (25.4%)	44 (34.9%)	
Marital status				
Married	167 (55.3%)	46 (15.2%)	89 (29.5%)	< 0.01
Unmarried	676 (44.9%)	282 (18.8%)	546 (36.3%)	
Whether have children				
Without	551 (45.1%)	221 (18.1%)	449 (36.8%)	0.11
Have	293 (49.8%)	107 (18.2%)	188 (32.0%)	
Whether to work part-time once				
Without	498 (44.7%)	214 (19.2%)	403 (36.1%)	0.06
Have	344 (50.2%)	112 (16.4%)	229 (33.4%)	

### Factors associated with burnout and career choice regret in the multivariate analysis

The multicollinearity analysis indicated there was no collinearity for burnout (Additional file 1). Table 4 summarizes the factors associated with burnout and career choice regret. Model 1, in which demographic variables were entered, showed factors independently associated with burnout were gender, postgraduate entrance examination score, hours worked or studied per week, marital status, and having children (all *P* <  0.05). Women were at higher risk for burnout than men (odds ratio [OR] = 0.74, 95% confidence interval [CI] 0.56–0.99). Married postgraduates (OR = 0.56, 95% CI 0.39–0.80) and those without children (OR = 0.55, 95% CI 0.40–0.77) were less likely to show symptoms of burnout. Model 2 (including career choice regret) showed career choice regret was independently associated with burnout (*P* <  0.05). In Model 3 (both demographic variables and career choice regret), being married and without children were protective factors. Career choice regret was the strongest risk factor for burnout (OR = 3.17, 95% CI 2.33–4.32).

**Table 4:** Burnout with associated factors^a^

Characteristics	Model 1^b^		Model 2^c^		Model 3^d^	
	OR (95% CI)	*P*	OR (95% CI)	*P*	OR (95% CI)	*P*
Gender						
Male	0.74 (0.56–0.99)	0.04				
Female	1 (Reference)				1 (Reference)	
Scores of postgraduate entrance examination						
< 300	0.81 (0.49–1.36)	0.43			0.68 (0.41–1.12)	0.13
300–330	1.60 (1.02–2.51)	0.04			1.40 (0.90–2.19)	0.14
330–360	1.09 (0.69–1.74)	0.71			0.98 (0.61–1.55)	0.92
> 360	1 (Reference)				1 (Reference)	
Hours worked or studied per week (h)						
< 35	0.47 (0.28–0.78)	< 0.01				
35–45	0.60 (0.41–0.86)	< 0.01				
45–55	0.58 (0.41–0.82)	< 0.01				
> 55	1 (Reference)					
Marital status						
Married	0.56 (0.39–0.80)	< 0.01			0.50 (0.35–0.71)	< 0.01
Unmarried	1 (Reference)				1 (Reference)	
Whether have children						
Without	0.55 (0.40–0.77)	< 0.01			0.58 (0.42–0.82)	< 0.01
Have	1 (Reference)				1 (Reference)	
Career choice regret						
With			3.22 (2.42–4.29)	< 0.01	3.17 (2.33–4.32)	< 0.01
Neutral			2.13 (1.49–3.04)	< 0.01	2.97 (1.95–4.54)	< 0.01
Without			1 (Reference)		1 (Reference)	

Abbreviations: *CI* = confidence interval, *OR* = odds ratio

^a^OR  < 1 indicates that it is a preventive factor of burnout, where as OR > 1 indicates that it is a risk factor of burnout

^b^In model 1, demographic characteristics was independent variables

^c^In model 2, career choice regret was the independent variables

^d^In model 3, both demographic variables and career choice regret were independent variables

Multiple logistic regression analysis showed postgraduates with shorter hours worked or studied per week (OR = 0.64, 95% CI 0.47–0.88) had a low risk for career choice regret, whereas postgraduates who were married (OR = 1.54, 95% CI 1.07–2.20) had a high risk. Being without symptoms of burnout (OR = 0.33, 95% CI 0.24–0.45) was also associated with a low risk for career choice regret (Table 5).

**Table 5:** Career choice regret with associated factor

Career choice regret ^a^	Variables	OR (95% CI)	*P* value
Neutral versus without career choice regret	Academic year		
	First-year, master’s degree	1.91 (0.64–5.69)	0.25
	Second-year, master’s degree	3.19 (1.10–9.26)	0.03
	Third-year, master’s degree	2.45 (0.85–7.07)	0.10
	First-year, doctor’s degree	2.66 (0.76–9.24)	0.13
	Second-year, doctor’s degree	1.69 (0.43–6.61)	0.45
	Third-year, doctor’s degree	1 (Reference)	
	Hours worked or studied per week (h)		
	< 35	0.84 (0.52–1.35)	0.47
	35–45	0.64 (0.47–0.88)	0.01
	45–55	0.66 (0.49–0.90)	0.01
	> 55	1 (Reference)	
	Marital status		
	Married	1.24 (1.07–2.20)	0.02
	Unmarried	1 (Reference)	
	Burnout		
	Without	0.33 (0.24–0.45)	< 0.01
	With	1 (Reference)	
With career choice regret versus without career choice regret	Hours worked or studied per week (h)		
	< 35	0.84 (0.52–1.35)	0.47
	35–45	0.64 (0.47–0.88)	< 0.01
	45–55	0.66 (0.49–0.90)	< 0.01
	> 55	1 (Reference)	
	Marital status		
	Married	1.54 (1.07–2.20)	0.02
	Unmarried	1 (Reference)	
	Burnout		
	Without	0.33 (0.24–0.45)	< 0.01
	With	1 (Reference)	

Abbreviations: *CI* = confidence interval, *OR* = odds ratio

^a^OR < 1 indicates that it is a preventive factor of career choice regret, where as OR > 1 indicates that it is a risk factor of career choice regret

Note: The reference category is: without career choice regret

## Discussion

This nationwide study used a questionnaire survey to assess burnout and career choice regret in Chinese neurology postgraduates. We found a high prevalence of burnout and career choice regret among participants, with burnout being closely related to career choice regret. To our knowledge, this study is the first to examine this issue in this population.

### Prevalence and predictors of burnout

The prevalence of burnout among Chinese neurology postgraduates was 83.6%, which was higher than reported for other professions worldwide. For example, most previous studies suggested that Chinese medical postgraduates showed moderate levels of burnout [22]. For other clinical postgraduates in different countries, reported burnout rates were 63% for internal medicine, 60% for ophthalmology, and 40% for general surgery [30]. The incidence of burnout among US medical students was 76% [11]. The high prevalence of burnout in our sample may be attributable to the complexity of diagnosis and treatment for neurological diseases and the high pressure and low career satisfaction associated with this specialty [20, 31]. Our study showed that working long hours was a major risk factor for burnout, which was consistent with previous findings [32]. However, a study involving Dutch residents showed that although their working hours were restricted to 48 h per week, many doctors still suffered from burnout, which suggests that factors other than working hours may also be important [33].

Model 3 showed career choice regret was the most important factor influencing burnout. A study in Europe found that burnout tended to affect feelings and attitudes towards work, rather than actual working time [34]. We found that postgraduates with burnout had a high risk for career choice regret, which was consistent with previous studies [13, 18, 19]. Previous studies demonstrated that turnover among doctors can be significantly reduced by intervening in burnout [3]. Therefore, more attention should be paid to career choice regret among postgraduates to reduce future losses of neurologists.

### Prevalence and predictors of career choice regret

Career choice regret is an attitude and belief that individuals have about their work. It reflects internal professional values and motivation, both of which determine career choices [35]. In this survey, nearly half of the participating postgraduates would not choose to be a doctor again, indicating that many neurology postgraduates were dissatisfied with their career choice. In addition, about one-quarter of participants had thought about dropping out at least once. A previous survey in the US found that approximately 11% of students have serious thoughts of dropping out of medical school each year [36]. The difference between that study and ours may be related to the medical environment. A previous study suggested that a poor medical environment affected medical students’ professional identity and had a significant impact on their career choices, which were particularly affected by long working hours, pressure from medical disputes, and violence against medical workers [35]. It can be speculated that a poor medical environment may increase the risk for career choice regret.

We found that sleep deprivation and family factors were important elements in career choice regret. A previous study also found that chronic sleep deprivation caused depression and anxiety, which affected attitudes and efficiency at work [37]. Family factors included family income, marital status, and whether the participant had children. Postgraduates with a low monthly family income were more likely to plan to become doctors, which may because doctors are well-paid comparing with other careers. Those who were married had a high risk for career choice regret, possibly because their heavy clinical workload may lead to conflict between work and family life, and strained relationships with their spouse or children. A study [23] suggested that career choice regret among neurology residents was influenced by work-family compatibility, which was consistent with our findings.

### Further measures

Preventing burnout among medical students and reducing career choice regret are important to ensure there are enough neurologists to meet global demand. These tasks call for active cooperation from the government, hospitals, and medical students. First, the government could reform the medical system and improve the medical environment, which may improve postgraduates’ career choices and enhance the social and economic status of doctors/postgraduates. Second, hospitals could facilitate the construction of healthy working environments (both physically and mentally) through improving working conditions, rationally allocating workers, and setting up career development planning for staff. Hospitals could also ensure staff has sufficient rest time, and pay attention to any psychological problems among postgraduates. Third, it is important for postgraduates to understand burnout correctly, improve their physical and psychological status, and pay attention to the balance of work and rest.

### Limitations

This study had a number of limitations. First, the survey did not use random sampling methods, which might have resulted in selection and response bias. However, the findings of this study may have some generalizability given the large and diverse sample. Second, only limited information was collected on demographic characteristics and other influencing factors, and burnout and career choice regret may be affected by other confounding variables. Third, the survey was cross-sectional, and we could not examine dynamic changes in burnout factors, which may be influenced by geography, professional factors, the local practice environment, and practice types. Further studies with more rigorous designs are needed to confirm these findings.

## Conclusion

Overall, symptoms of burnout and career choice regret are prevalent among Chinese neurology postgraduates. Career choice regret is the strongest predictor of burnout in this population. Our results provide a high-level overview to support interventions to prevent burnout and improve career choice regret. Active measures from the government, hospitals, and postgraduates are needed to change the current situation.

## Additional file

Additional file 1: The initial and final logistic models of binary logistic regression analysis of burnout. (XLS 41 kb)
